# Editorial: Presence and Daily Exposure to Endocrine Disruptors: How Can Human Life Change?

**DOI:** 10.3389/fendo.2021.790853

**Published:** 2021-11-24

**Authors:** Elena Grasselli, Marketa Dvorakova, Jones B. Graceli

**Affiliations:** ^1^ Department of Earth, Environmental and Life Sciences (DISTAV), University of Genoa, Genoa, Italy; ^2^ National Reference Laboratory for Experimental Immunotoxicology, Centre of Toxicology and Health Safety, National Institute of Public Health (NIPH), Prague, Czechia; ^3^ Department of Morphology, Federal University of Espírito Santo, Vitória, Brazil

**Keywords:** endocrine disrupting chemicals, daily exposure, personal care, cosmetics, food, water, animal models, *in vitro* tests

An endocrine-disrupting chemical (EDC) is defined by the Endocrine Society and the World Health Organization (WHO) as “an exogenous substance or mixture that alters function(s) of the endocrine system and consequently causes adverse health effects in an intact organism, or its progeny, or (sub)populations” (1, 2). The structural similarities between EDCs and endogenous hormones lead to EDCs being able to interfere with the regular function of hormone receptors (3). ECDs are able to act at any stage of the human lifespan, causing several devastating effects on the health of individuals, such as obesity, cancer, and reproductive malformation and/or improper function (Bokobza et al.; Komarowska et al.; Rodprasert et al.; Zhang et al.), eventually leading to death, dependent on the type of EDC, dose, period, time of EDC exposure, or if there was an EDC mixed exposure (1, 2).

This Research Topic reported on techniques to study reproductive and metabolic disorders, such as use of magnetic resonance imaging (MRI) to study polycystic ovary syndrome (PCOS) (Li et al.). They demonstrated that cognitive impairment and emotional changes are associated with high serum testosterone levels, luteinizing hormone and fasting insulin levels in women with PCOS (Li et al.). *In vitro* fertilization and embryo transfer (IVF-ET) was suggested to improve complications in spontaneous ovarian hyperstimulation syndrome caused by pituitary adenoma secreting follicle-stimulating hormone (Du et al.).

In addition, this Research Topic showed an important link between alterations of reproductive and metabolic health as a result of EDC exposure, such as bisphenol A, bisphenol S, bisphenol F, phthalate metabolites, and mercury (3; Bokobza et al.; Komarowska et al.; Rodprasert et al.). Specifically, uterine leiomyomata and endometriosis development were associated with urinary phthalate metabolites and serum mercury levels from a female cohort from the National Health and Nutrition Examination Survey (NHANES, 2001-2006). Komarowska et al. provided a first characterization of prepubertal boys suffering from cryptorchidism who were exposed to different kinds of bisphenols (BP) from the Pediatric Surgery and Urology Department, Medical University of Bialystok (2017 - 2018). They suggest that cryptorchid boys are widely exposed *in utero* with high blood bisphenol A levels and, to a lesser extent, also to its alternatives, such as bisphenol S and F. Hypospadias and decreased anogenital distance frequency were also associated with BP exposure (Komarowska et al.).

Another review study has shown that metabolic EDCs exposure (such as bisphenol A, phthalates, dichlorodi-phenyldichloroethylene, dioxins, polychlorinated biphenyls, organochlorine pesticides, polybrominated flame retardants and perfluorinated compounds, etc.) act as obesogenic chemicals, leading to metabolic abnormalities, impairing normal adipose tissue function, increasing adipose tissue mass, remodeling, hypoxia, and inflammation, with the prevalence of non-monotonic dose-response curves (1, 2; Bokobza et al.). The abnormal metabolic adipose tissue function, as a result of EDC exposure, alters adipokines’ profile release and hormone actions (1, 2; Bokobza et al.). It is now well established that abnormal adipose tissue secretes adipocytokines that could help to promote tumor progression. In parallel, EDCs exposure has been implicated in the development of cancers, in particular hormone-dependent cancers (such as prostate, testis, breast, endometrium, and thyroid) (Bokobza et al.).

This Research Topic brings together six papers (four original research and two review articles) on endocrine and metabolic disorders, such as polycystic ovary syndrome (PCOS) and pituitary adenoma secreting follicle-stimulating hormone. In addition, the topic reported a complex toxicologic role of endocrine disruption chemical (EDC) exposure, such as bisphenol A (BPA), bisphenol S (BPS), and bisphenol F (BPF), phthalate metabolites, mercury, etc, linked with human health complications such as cancer, obesity, and reproductive abnormalities. Understanding the interplay between some EDCs, as well as their exposure from different sources and physiologic abnormalities, is highly relevant for human health in the world. Clearly, research in this field is advancing at a rapid pace. The articles in this Research Topic highlight novel findings and unanswered questions for future investigation.

## Author Contributions

EG conceived the topic. MD helped to manage. JG shared his previous experience and collaborated to the editorial process. All authors contributed to the article and approved the submitted version.

## Funding

This research was supported by CNPq (#304724/2017-3/N° 12/2017), FAPES/CNPq (PRONEX, #24/2018). JG was awarded grants by FAPES and CNPq. This research was supported by AngelConsutling s.r.l.

## Conflict of Interest

The authors declare that the research was conducted in the absence of any commercial or financial relationships that could be construed as a potential conflict of interest.

## Publisher’s Note

All claims expressed in this article are solely those of the authors and do not necessarily represent those of their affiliated organizations, or those of the publisher, the editors and the reviewers. Any product that may be evaluated in this article, or claim that may be made by its manufacturer, is not guaranteed or endorsed by the publisher.
